# Micro-Stick to Rapid Infusion Catheter (RIC): Ultrasound-Guided Rapid Infusion Catheter Insertion Using a Micropuncture Kit

**DOI:** 10.7759/cureus.35813

**Published:** 2023-03-06

**Authors:** Philip L Stagg

**Affiliations:** 1 Faculty of Health Sciences and Medicine, Bond University, Gold Coast, AUS; 2 Anaesthetics, Gold Coast Private Hospital, Gold Coast, AUS

**Keywords:** seldinger technique, ultrasound-guided vascular access, difficult intravenous access, rapid infusion catheter, micropuncture kit

## Abstract

Ultrasound-guidance improves success for difficult intravenous access (DIVA), particularly when difficulty is anticipated. However, insertion of a wide-bore sheath, such as a rapid infusion catheter, is likely to pose additional problems and unique challenges in this context, despite ultrasound guidance. With the aid of a video clinical simulation, this article describes an ultrasound-guided technique for inserting a proprietary rapid infusion catheter (RIC) or similar wide-bore sheath using the micropuncture kit.

## Introduction

Peripheral intravenous access occurs in up to 80% of hospitalized patients [1] and is a procedure that is vital for a broad range of diagnostic and therapeutic interventions. Unfortunately, difficult intravenous access (DIVA) is a common occurrence, with a negative impact on treatment and patient experience. Witting et al. found that DIVA occurred in 39% of patients in an urban emergency department [2]. Furthermore, DIVA requiring attempts by multiple care providers occurred in 28% of cases and resulted in treatment delays of up to 120 mins in 5% of patients. From the patient’s perspective, intravenous access is painful [2]. While there is no universally accepted definition, DIVA can be defined as two or more failed attempts at intravenous access [3] or the requirement of a more experienced care provider [2]. Predictors of DIVA include a history of DIVA and the absence of visible or palpable veins on physical examination [4]. Prediction of DIVA is likely to enable physicians to customize treatment and facilitate forward planning [2]. 

Real-time ultrasound guidance improves cannulation success rates for difficult intravenous access [5]. In addition, it reduces the number of punctures, reduces time, and improves patient satisfaction, albeit without a reduction in complications [5]. International guidelines suggest that ultrasound guidance should be considered early for any type of peripheral intravenous access device when difficult intravenous access is anticipated or encountered [6]. 

The rapid infusion catheter (RIC®) (Arrow® International, Morrisville, NC, USA) is indicated for rapid volume infusion [7]. Insertion typically requires the exchange of a 20-Ga or larger peripheral intravenous catheter to the RIC® sheath [7]. However, guidance regarding how best to insert a RIC® in the context of DIVA is lacking. 

Conversely, micropuncture kits such as the Micropuncture® Access Set (Cook Medical, Bloomington, Indiana, USA) and V•Stick™ Vascular Access Set (Argon Medical, Plano, Texas, USA) may be useful for ultrasound-guided DIVA [8-10]. Micropuncture kits typically contain a 21-Ga introducer needle, a 0.018-inch guidewire, and a 4-Fr to 5-Fr 10 cm introducer catheter. After inserting a micropuncture catheter, a larger wire of up to 0.038-inch diameter can be advanced through the micropuncture catheter. After removing the introducer catheter and leaving the larger wire in situ, a larger sheath (usually interventional) can be advanced over the wire and into the vessel [11]. In this manner, the micropuncture catheter can be exchanged for a range of coaxial-dilator-mounted percutaneous sheaths. 

This article describes an ultrasound-guided technique for the insertion of a RIC using a micropuncture kit in the context of difficult peripheral venous access. This method of securing a RIC is well suited to DIVA, where large-bore peripheral vascular access is required for volume resuscitation. 

## Technical report

Video 1 demonstrates the ultrasound-guided technique for the insertion of a RIC using a micropuncture kit in the context of difficult peripheral venous access (Video 1). A VATA 2365 - Advanced Venipuncture Training Aid ™ (VATA, Canby, Oregon, USA) is used in the clinical simulation. 

**Video 1:** Ultrasound-guided rapid infusion catheter (RIC) insertion using a micropuncture kit. Under real-time ultrasound guidance, a 21-Ga 7 cm introducer needle with an echogenic tip is inserted into the target vessel. A 0.018-inch 40 cm guidewire is inserted through the introducer needle, and the needle is removed. After ultrasound confirmation that the wire is correctly located within the target vessel, the 4-Fr (18-Ga) or 5-Fr (16-Ga) 10 cm micropuncture introducer catheter with the coaxial dilator is advanced into the vessel. Removal of the inner dilator and guidewire facilitates insertion of the 0.025-inch (0.64 mm) 33 cm rapid infusion catheter (RIC) wire through the micropuncture introducer catheter. A small, 2-to-3 mm skin incision with an #11 scalpel blade facilitates insertion of the 8.5-Fr 6.4 cm RIC catheter with tissue dilator. The tissue dilator and wire are removed as a unit. The RIC is attached directly to the intravenous fluid giving set, avoiding needle-free connectors or other intermediaries that may reduce flow rates. The RIC is stitched in if required, and a transparent dressing is applied. In this video, the RIC catheter is attached to Belmont® Rapid Infuser RI-2 (Billerica, MA, United States) infusion tubing. A VATA 2365 – Advanced Venipuncture Training Aid ™ (VATA, Canby, Oregon, USA) is used in this clinical simulation.

Instructions for ultrasound-guided RIC insertion using a micropuncture kit, as used in this proof-of-concept experiment, are as follows: using a strict aseptic technique, and after applying the tourniquet, infiltrating the skin with a local anesthetic. Under real-time ultrasound guidance, insert the echogenic 21-Ga 7 cm introducer needle. Advance the fine 0.018-inch (0.46 mm) 40 cm micropuncture guidewire through the introducer needle, and withdraw the introducer needle, leaving the guidewire in place. Confirm with ultrasound that the guidewire is within the target vessel. Advance the 4-Fr (18-Ga) or 5-Fr (16-Ga) 10 cm micropuncture introducer catheter and coaxial dilator over the guidewire and into the vessel while applying gentle skin traction. Remove the dilator and guidewire, leaving the introducer catheter in place. Advance the 0.025-inch (0.64 mm) 33 cm RIC guidewire through the introducer catheter and remove the introducer catheter leaving the RIC guidewire in place. Load the 8.5-Fr 6.4 cm RIC sheath and coaxial dilator onto the guidewire, ensuring that the guidewire exits the catheter hub. Make a 2-3 mm superficial skin nick with an #11 scalpel blade. Applying skin traction, advance the RIC sheath and dilator together as a unit until the catheter is part-way into the target vessel. Advance the RIC catheter off the dilator and the rest of the way into the vessel. Remove the inner dilator and wire and attach directly to intravenous administration tubing, avoiding needle-free connectors or other intermediaries that may reduce flow rates. Stitch in the catheter if required, and cover with a transparent dressing. 

**Figure 1:**
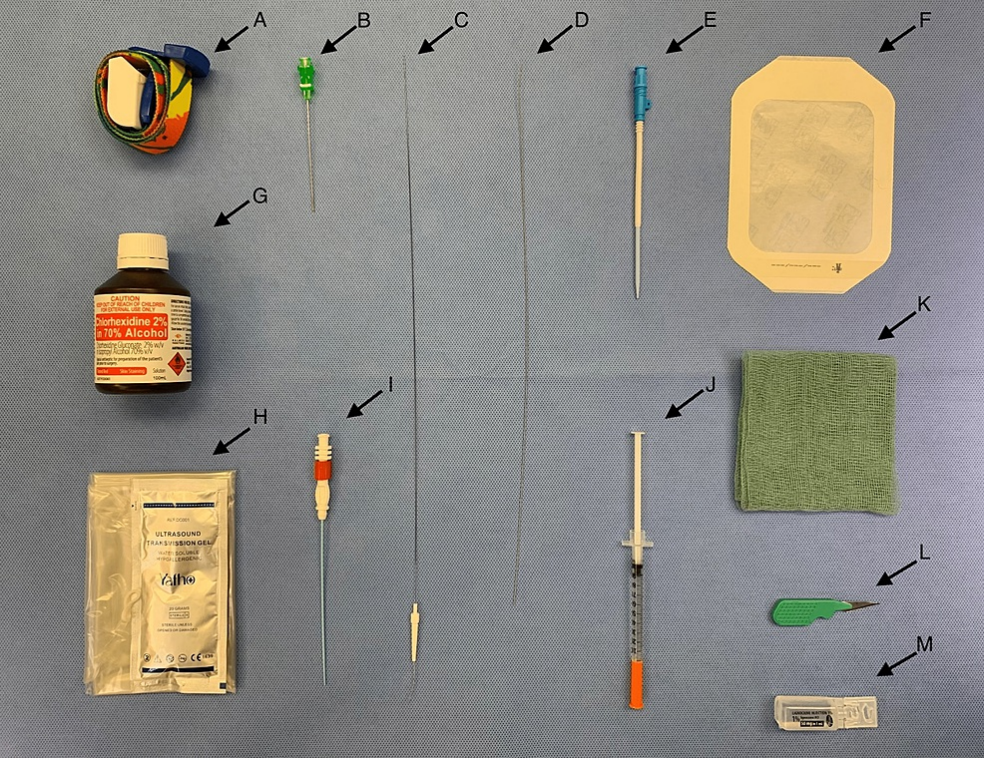
Equipment required for ultrasound-guided insertion of a micropuncture introducer catheter and conversion to a rapid infusion catheter (RIC). (A) Tourniquet. (B) 21-Ga 7 cm introducer needle with echogenic tip. (C) 0.018-inch diameter, 40 cm nitinol guidewire with floppy radiopaque tip. (D) Rapid infusion catheter (RIC®) 0.025-inch diameter, 13-1/8-inch (33 cm) spring-wire guide.  (E) 8.5-Fr 2-½-inch (6.4 cm) Arrow-Flex® radiopaque RIC® catheter with tissue dilator. (F) Large transparent dressing. (G) Chlorhexidine 2% in 70% alcohol antiseptic skin preparation solution. (H) Ultrasound probe cover. (I) 4-Fr micropuncture introducer catheter with a coaxial dilator. (J) Insulin syringe for local anesthetic for skin infiltration. (K) Gauze. (L) #11 scalpel. (M) 1% lignocaine. (N.B. While an Argon Medical V•Stick™ Vascular Access Set is used in this image, however, a Cook® Micropuncture® Access Set can also be used).

Figure 1 shows the equipment required for ultrasound-guided insertion of a RIC using a micropuncture kit. 

**Figure 2:**
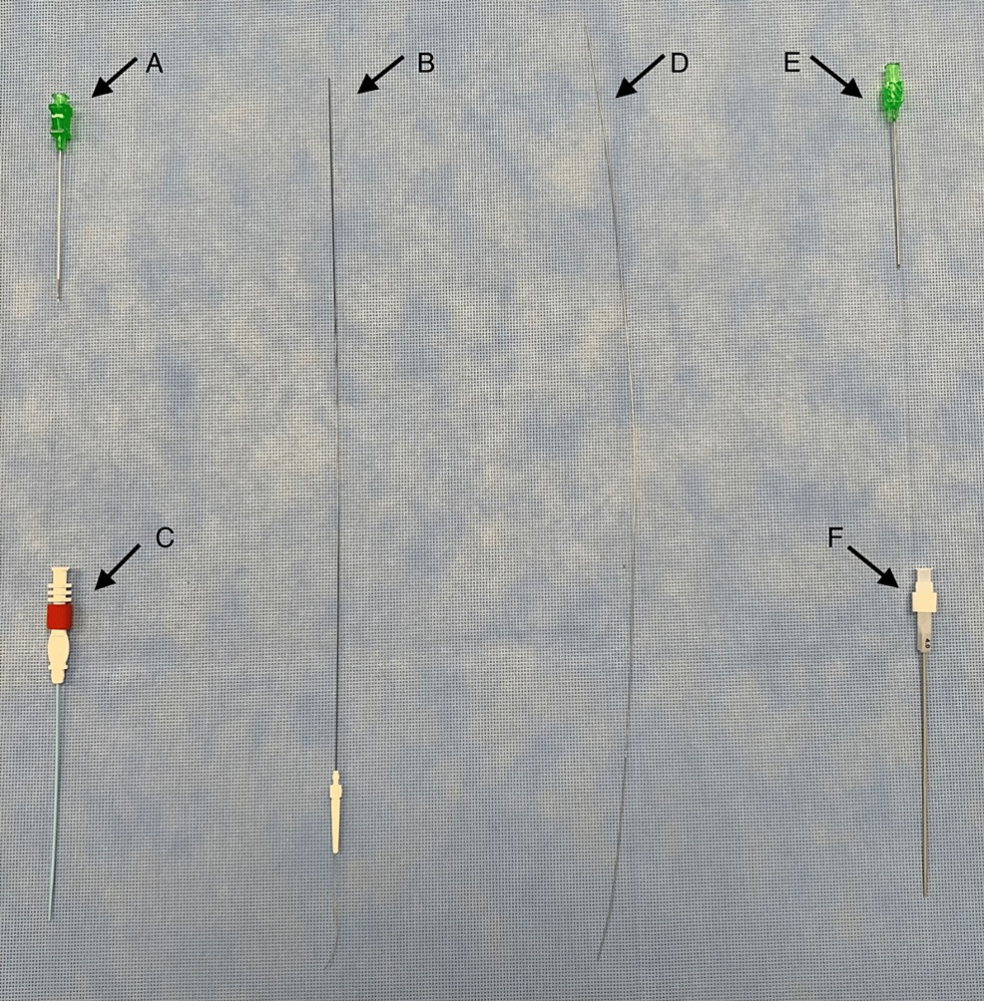
Comparison of an Argon Medical V•Stick™ Vascular Access Set and a Cook® Micropuncture® Access Set. (A) to (C): V•Stick™ Vascular Access Set 4-Fr (Ref: 391504300E). (A) V•Stick™ 21-Ga UltraSharp™ 7 cm introducer needle with echogenic tip. Note that the textured, echogenic tip is a prominent feature and circumferential. The ultra-sharp tip is designed to reduce drag and ease insertion. (B) V•Stick™ 0.018-inch 40 cm nitinol guidewire with floppy radiopaque tip. Note the presence of a plastic guide to aid the insertion of the wire into the hilt of the introducer needle. Nitinol has shape memory and resistance to bending, accounting for the straightness of the wire in the photo. The floppy tip is atraumatic and allows flexibility during placement (available also in stainless steel).  (C) V•Stick™ 4-Fr 10 cm SmoothTrans ™ introducer catheter with a coaxial dilator. The red locking nut indicates a standard stiffness catheter and dilator but is also available in extra stiff composition. (D) to (E): 4-Fr Cook® Micropuncture® Access Set Silhouette®  Transitionless Push-Plus ™ Design – Stiffened Cannula (Ref: G48007). (D) Cook® 0.018-inch 40 cm stainless steel Torq-Flex® guidewire with floppy tip. Stainless steel increases the stiffness of the guidewire, giving good torque control, but is easier to kink. Fewer flexibility accounts for the curved shape of the wire in the image (available also in nitinol). (E) Cook® 21-Ga 7 cm introducer needle with echogenic tip. Note that the textured echogenic tip is subtler and located on the beveled surface. (F) Cook® 4-Fr 10 cm introducer catheter with Silhouette Transitionless™ coaxial dilator.

A micropuncture kit typically contains a 21-Ga 7 cm echogenic needle, a 40 cm 0.018-inch (0.46 mm) guidewire, and a 10 cm 4-Fr or 5-Fr sheath with a coaxial dilator. Figure 2 shows the contents of an Argon V•Stick™ Vascular Access Set and a Cook® Micropuncture® Access Set side by side for comparison. 

## Discussion

Using clinical simulation and video multimedia, this paper presents a novel ultrasound-guided technique for the insertion of a RIC using a micropuncture kit for difficult peripheral venous access. This simulation provides proof of concept that a micropuncture kit may be used to facilitate the insertion of a RIC, or similar wide-bore sheaths, for clinical situations requiring volume resuscitation in patients with known or anticipated DIVA. I have used this technique for several major surgical procedures without complication, with high levels of patient satisfaction, using the micropuncture sheath to facilitate the insertion of similar large sheaths (e.g., 6-Fr sheaths) for volume resuscitation. The principal advantage of this method is that both real-time ultrasound guidance and the classic Seldinger technique, upon which this technique is based, have been shown to increase vascular access success in difficult patient populations [5,12]. Furthermore, ultrasound guidance is recommended by international guidelines when difficult peripheral access is anticipated or encountered [6]. An additional potential advantage of this technique is that where time permits, it may obviate the need for other more invasive DIVA solutions, such as central venous and intra-osseous (IO) access, and their inherent risks. Notwithstanding, additional studies will be required to further elucidate the benefits and pitfalls of this technique before it can be recommended for routine practice in such patients.

Whilst more commonly used by interventional radiologists, vascular surgeons, and cardiologists, the potential role of the micropuncture kit in DIVA is being increasingly recognized in anesthesia and critical care and has been utilized in somewhat nuanced and creative ways [8-10]. Castillo et al. presented a case of a 30 cm 16-Ga central venous catheter being inserted into the axillary vein with the aid of a 4-Fr micropuncture kit in a patient with severe musculoskeletal deformity where neck veins could not be accessed, thus prohibiting conventional central line insertion [8]. Bowman and Neto report the practice of loading a 14-Ga cannula onto the 5-Fr micropuncture sheath and inner dilator and using the micropuncture sheath as a coaxial introducer as a means of smoothly inserting a 14-Ga cannula [9]. Montrief et al. discuss the micropuncture kit as a solution for difficult vascular access, highlighting possible benefits and potentially reduced complications [10]. 

Despite the potential advantages of this technique, when utilizing micropuncture kits for the insertion of a RIC under ultrasound guidance, anatomical considerations such as vessel depth and diameter are necessary. In a prospective study, Witting et al. found that ultrasound-guided cannulation success was primarily dependent on vessel characteristics [13]. Success rates were significantly higher when vessel depth was between 3-15 mm and when vessel diameter was more than 4 mm. Regarding catheter survival, Bahl et al. found that greater than 2.75 cm of the catheter should dwell within the vessel to optimize catheter dwell time [14]. However, there is a lack of guidance in the literature regarding appropriate target vessel selection for the placement of a RIC, with the product information instructions being a notable omission, simply stating that a 20-Ga cannula is the requisite requirement [7]. Online emergency medicine resources suggest that optimal vessels for RIC insertion include cephalic or basilic veins in the cubital fossa or forearm, the saphenous vein, or the external jugular vein, ideally with a straight 6-to-8 cm stretch of vein [15]. Taken together, the body of evidence indicates that careful vessel selection with an ultrasound pre-scan is essential when embarking on this technique. Further, obese patients with deeper veins that are neither visible nor palpable, yet are of good caliber, are one subgroup in which this technique is likely to be useful. 

Listed potential complications for the micropuncture sheath and the RIC include vessel wall perforation, infiltration/extravasation, catheter or wire embolism, infection, thrombosis, inadvertent arterial puncture, nerve damage, hematoma, intravascular clotting, necrosis, scarring, pain, and hemorrhage [7,11].

However, the use of a micropuncture kit has the potential to reduce complications. A retrospective cohort of 17,844 patients undergoing percutaneous coronary intervention via the femoral artery demonstrated a significantly lower rate of groin hematoma and a significantly lower complication rate overall using the 21-G micropuncture needle compared with the standard 18-G needle [16]. There was no significant difference in the rate of limb ischemia, vascular perforation, pseudoaneurysm, retroperitoneal bleeding, and arteriovenous fistula. Notwithstanding, micropuncture complication rates when used for central or peripheral venous access have not been formally elucidated, nor have success rates or patient satisfaction; this is a much-needed area for future study. Indeed, touted risk reduction associated with micropuncture kits is largely due to the use of a small-bore access needle and not due to the design of the catheter itself. These catheters tend to be relatively stiff and thus carry a theoretical risk of damage to small peripheral vessels that is yet to be quantified. 

Reported RIC complications are rare. In a retrospective analysis of 839 patients who underwent RIC placement for a liver transplant, a low 1.67% incidence of complications was observed [17]. Of the 14 patients with RIC-related complications, three had hematoma formation requiring surgical intervention, while the rest were managed conservatively. However, rare, serious complications such as compartment syndrome and skin necrosis have occurred [18]. Thus, while the micropuncture kit offers a solution for difficult peripheral access and affords the opportunity to upsize, these risks, along with other potential challenges, such as the loss of peripheral vessel integrity during the exchange, must always be considered when attempting this technique. *** IO careful consideration of the risks, benefits, and alternatives is warranted when embarking on this technique as a DIVA solution. 

A comparison of the two common micropuncture kits - the Argon Medical V•Stick™ Vascular Access Set and a Cook® Micropuncture® Access Set revealed similarities and subtle differences. The echogenic tip of the V•Stick™ UltraSharp™ introducer needle is an obvious feature, being circumferential and quite prominent. Conversely, the echogenic tip of the Cook® introducer needle seemed subtler and was located on the beveled surface only in the kit used for this experiment. The V•Stick™ claims to have an ultra-sharp tip designed to reduce drag and ease insertion [19]. I have found both Cook® and V•Stick™ introducer needles to be excellent for DIVA, with adequate sharpness and excellent echogenicity. However, a unique aspect of using the 21-Ga 7 cm introducer needle for peripheral intravenous access with this technique is that blood return is typically sluggish and delayed compared to 18-Ga central line access needles. This is due to both the finer gauge and the longer length of the introducer needle. This can surprise inexperienced users and elevates the importance of ultrasound for needle placement when using this technique. Both Cook® and V•Stick™ kits have the option of a nitinol or stainless-steel guidewire with a floppy radiopaque tip. Nitinol has shape memory (accounting for the straightness of the wire in the photo) and kink resistance, providing more control during insertion [10]. The floppy tip is atraumatic and allows flexibility during placement [10,20]. The floppy tip was more noticeable in the V•Stick™ kit used in this experiment. The presence of a plastic guide to aid the insertion of the wire into the hilt of the introducer needle is a useful feature of the V•Stick™. Note that nitinol contains nickel, a common cause of allergic contact dermatitis, so the use of a kit with a stainless-steel wire would seem prudent in such patients. Insertion of the micropuncture introducer catheter was smooth and seamless for both the Cook® and V•Stick™ kits due to the Cook® Transitionless-Tip™ & V•Stick™ SmoothTrans™ coaxial introducers, which have been designed to glide into the vessel with less force [19,20]. Both kits are available in standard and extra-stiff compositions, depending on user preference. 

The main limitation of this study is that it has described a novel technique for inserting a RIC in the context of DIVA, ex-vivo, using a clinical simulation and video multimedia. As such, it should only be considered proof of concept for the validity of the technique. In addition, this technique requires a number of procedural steps, and is primarily intended for practitioners adept in ultrasound-guided vascular access, so would not be practical for those less experienced in ultrasound-guided peripheral venous access. As such, adequate training and competency assessment would be prudent before the implementation of this technique by such practitioners. While this technique could be used for electives or emergencies requiring wide-bore access where time permits, in true time-critical emergencies in patients with DIVA, it would be inappropriate. In such high-stakes emergencies, IO or central venous access would be more appropriate. Notwithstanding these limitations, I have used this technique to insert a range of wide-bore sheaths in my clinical practice, predominantly for elective anesthesia where there is the potential for major volume resuscitation, with high levels of success and patient satisfaction. Further studies are now required to quantify the benefits and limitations of this technique, including success rates, time to RIC insertion, reductions in theatre delays, complication rates, and patient satisfaction and comfort. 

## Conclusions

There is an emerging role for the micropuncture kit in the field of difficult intravenous access requiring ultrasound guidance. The unique features of an echogenic introducer needle and an easy-to-insert micropuncture introducer catheter that can accommodate a wire up to 0.038-inches in diameter provide a unique opportunity for practitioners to upsize to a variety of wide-bore sheaths for fluid resuscitation in patients with DIVA. This paper provides proof of concept that a micropuncture kit can be used to facilitate the insertion of a RIC using a real-time ultrasound-guided technique for DIVA. Further studies will be now required to further elucidate the efficacy, complications, and patient and user satisfaction with this technique. 
